# Probiotics Prevent Late-Onset Sepsis in Human Milk-Fed, Very Low Birth Weight Preterm Infants: Systematic Review and Meta-Analysis

**DOI:** 10.3390/nu9080904

**Published:** 2017-08-22

**Authors:** Arianna Aceti, Luca Maggio, Isadora Beghetti, Davide Gori, Giovanni Barone, Maria Luisa Callegari, Maria Pia Fantini, Flavia Indrio, Fabio Meneghin, Lorenzo Morelli, Gianvincenzo Zuccotti, Luigi Corvaglia

**Affiliations:** 1 Neonatology and Neonatal Intensive Care Unit, Department of Medical and Surgical Sciences (DIMEC), University of Bologna, S.Orsola-Malpighi Hospital, 40138 Bologna, Italy; arianna.aceti2@unibo.it (A.A.); i.beghetti@gmail.com (I.B.); 2 Neonatal Unit, Catholic University, 00168 Rome, Italy; luca.maggio@fastwebnet.it (L.M.); gbarone85@yahoo.it (G.B.); 3 Department of Biomedical and Neuromotor Sciences (DIBINEM), University of Bologna, 40126 Bologna, Italy; dedegori27@gmail.com (D.G.); mariapia.fantini@unibo.it (M.P.F.); 4 Institute of Microbiology, Catholic University, 29122 Piacenza, Italy; marialuisa.callegari@unicatt.it (M.L.C.); lorenzo.morelli@unicatt.it (L.M.); 5 Department of Pediatrics, Aldo Moro University, 70124 Bari, Italy; f.indrio@alice.it; 6 Division of Neonatology, V. Buzzi Children Hospital, 20154 Milan, Italy; fabio.meneghin@icp.mi.it; 7 Department of Pediatrics, V. Buzzi Children Hospital, University of Milan, 20154 Milan, Italy; gianvincenzo.zuccotti@unimi.it

**Keywords:** late-onset sepsis, probiotic, preterm infants, human milk, meta-analysis

## Abstract

Growing evidence supports the role of probiotics in reducing the risk of necrotizing enterocolitis, time to achieve full enteral feeding, and late-onset sepsis (LOS) in preterm infants. As reported for several neonatal clinical outcomes, recent data have suggested that nutrition might affect probiotics’ efficacy. Nevertheless, the currently available literature does not explore the relationship between LOS prevention and type of feeding in preterm infants receiving probiotics. Thus, the aim of this systematic review and meta-analysis was to evaluate the effect of probiotics for LOS prevention in preterm infants according to type of feeding (exclusive human milk (HM) vs. exclusive formula or mixed feeding). Randomized-controlled trials involving preterm infants receiving probiotics and reporting on LOS were included in the systematic review. Only trials reporting on outcome according to feeding type were included in the meta-analysis. Fixed-effects models were used and random-effects models were used when significant heterogeneity was found. The results were expressed as risk ratio (RR) with 95% confidence interval (CI). Twenty-five studies were included in the meta-analysis. Overall, probiotic supplementation resulted in a significantly lower incidence of LOS (RR 0.79 (95% CI 0.71–0.88), *p* < 0.0001). According to feeding type, the beneficial effect of probiotics was confirmed only in exclusively HM-fed preterm infants (RR 0.75 (95% CI 0.65–0.86), *p* < 0.0001). Among HM-fed infants, only probiotic mixtures, and not single-strain products, were effective in reducing LOS incidence (RR 0.68 (95% CI 0.57–0.80) *p* < 0.00001). The results of the present meta-analysis show that probiotics reduce LOS incidence in exclusively HM-fed preterm infants. Further efforts are required to clarify the relationship between probiotics supplementation, HM, and feeding practices in preterm infants.

## 1. Introduction

Late onset sepsis (LOS) is one of the most common causes of morbidity and mortality in preterm infants [1,2]. It occurs in approximately 20% of very low birth weight (VLBW) infants, has a significant overall mortality [3], and a high risk of long-term neurodevelopmental sequelae [4].

Beyond an immature skin-mucosal barrier and immune response, other well-recognized risk factors for LOS include long-term use of invasive interventions, failure of early enteral feeding with breast milk, prolonged duration of parenteral nutrition, hospitalization, surgery, and underlying respiratory and cardiovascular diseases [2].

Growing evidence supports the key role of a healthy gut microbiota in promoting and maintaining a balanced immune response and in the establishment of the gut barrier in the immediate postnatal life [5]. However, in preterm infants, the development of the microbial community is disrupted by events related to prematurity: Mode of delivery, antenatal and postnatal use of antibiotics, minimal exposure to maternal flora, and low intake of breast milk [6]. Such disruption, called dysbiosis, results in an altered barrier and immune function and an imbalance between pro- and anti-inflammatory responses, and has been associated with necrotizing enterocolitis (NEC) and LOS [7,8].

Probiotics, defined as live micro-organisms that confer health benefits to the host through an interaction with gut microbiota and immune function when administered at adequate doses [9], have been proposed as potential tools to prevent NEC and LOS [10].

Updated meta-analyses confirm the benefits of probiotics in reducing the risk of NEC [11,12], the time to achieve full enteral feeding [13,14], and the risk of LOS [15,16] in preterm infants. However, most of these meta-analyses fail to explore the role of probiotics in deeper detail, and do not provide specific recommendations regarding which probiotic strain or mixture of strains should be used, and which population would benefit most from the use of probiotics. 

Gut colonization in human milk (HM)-fed preterm infants is different from that of formula-fed infants [17]. HM provides nutrients, prebiotic carbohydrates, endogenous probiotics, and a variety of bioactive factors that exert beneficial effects directly and indirectly on host-gut microbiota interactions [18]. Recent data suggest that probiotic efficacy might be dependent upon the type of feeding; specifically, only preterm infants receiving HM would benefit from probiotic use in terms of a lower risk of NEC [19] and a reduction in the time needed to achieve full enteral feeding [13]. Furthermore, in vitro studies have shown that the growth of some probiotic species is enhanced in the presence of HM oligosaccharides (HMOs) [20,21]. Despite these suggestions, however, only a few randomized controlled trials (RCTs) report the type of feeding in infants given probiotics; and also for this reason, meta-analyses are unable to make any consideration about the influence of type of feeding in reducing adverse outcomes, such as NEC or LOS, in preterm infants receiving probiotics [13,16].

The aim of this systematic review and meta-analysis is thus to evaluate the effect of probiotics for the prevention of LOS in preterm infants according to type of feeding (exclusive HM vs. exclusive formula or mixed feeding).

## 2. Materials and Methods

### 2.1. Literature Search

The study protocol was designed by the members of the Task Force on Probiotics of the Italian Society of Neonatology. A systematic review of published studies reporting the use of probiotics for the prevention of LOS in preterm infants, according to type of feeding, was performed in accordance with PRISMA guidelines [22].

The characteristics of the studies included in the systematic review were the following: Randomized and quasi-randomized controlled trials involving preterm infants (gestational age (GA) <37 weeks) who had received, within one month of age, any probiotic compared to placebo or no treatment. The outcome of interest was culture-proven LOS, defined as the presence of a positive blood or cerebrospinal fluid culture taken >72 h after birth.

PubMed (http://www.ncbi.nlm.nih.gov/pubmed/), the Cochrane Library (http://www.cochranelibrary.com/) and Embase (http://www.embase.com/) were interrogated for studies published before 28 October 2016. The following string was used to perform the PubMed search: ((infant OR infants) OR (neonate OR neonates) OR (newborn OR newborns) AND (septi* OR sepsi* OR sepsis) OR (bacterial infect* OR bacterial infections (MH)) AND (probiotic OR probiotics OR pro-biotic OR pro-biotics)) NOT (animals (MH) NOT humans (MH)). The string was built up by combining all the terms related to LOS and probiotics, using PubMed MeSH terms, free-text words, and their combinations through the most proper Boolean operators, in order to be as comprehensive as possible. Similar criteria were used for searching the Cochrane Library and Embase. The review was restricted to English-written studies involving human subjects.

Luca Maggio (LM), Giovanni Barone (GB), Arianna Aceti (AA), and Isadora Beghetti (IB) performed the literature search. Potentially eligible studies were identified from the abstracts; the full texts of relevant studies were assessed for inclusion and their reference lists were searched for additional studies.

### 2.2. Data Extraction and Meta-Analysis

Study details (population, characteristics of probiotic and placebo, type of feeding, and outcome assessment) were evaluated independently by LM, GB, AA, and IB, and checked by Davide Gori (DG). Study quality was evaluated independently by AA, IB, and DG using the risk of bias tool as proposed by the Cochrane collaboration (Chapter 8 of the Cochrane Handbook of Systematic Reviews) [23]. In addition, an assessment of the quality of evidence using the GRADE working group approach was performed [23]. The evaluation was carried out by DG following Chapter 12 of the Cochrane Handbook [23] and classifying the evidence as high, moderate, low, and very low (as suggested by the GRADE Working Group) [24].

When outcome data were not reported according to type of feeding, the corresponding authors of the papers were contacted by email and were asked to provide separate data for LOS incidence in infants receiving probiotics vs. placebo according to type of feeding (exclusive HM vs. exclusive formula or mixed feeding). If the corresponding author was unable to provide these data or did not reply to the email, the paper was excluded from the meta-analysis.

The association between probiotic use and LOS was evaluated by a meta-analysis conducted by AA, IB, and DG using the RevMan software (version 5.3, downloaded on 1 November 2016 from the Cochrane website: http://tech.cochrane.org/revman/download). Risk ratio (RR) was calculated using the Mantel–Haenszel method and reported with a 95% confidence interval (CI). A fixed-effect model was used for the analyses. Heterogeneity was assessed using the *χ*^2^ test and *I*^2^ statistic: If significant heterogeneity was found (*p* < 0.05 from the *χ*^2^ test) or the number of studies was lower than five, a random-effects model was used instead [23].

The results of the meta-analysis were presented using forest plots, while a funnel plot was used for investigating publication bias.

## 3. Results

### 3.1. Literature Search

The number of potentially relevant papers identified through the literature search was 2713 (1401 in PubMed, 83 in the Cochrane Library, and 1229 in Embase).

**Figure 1:**
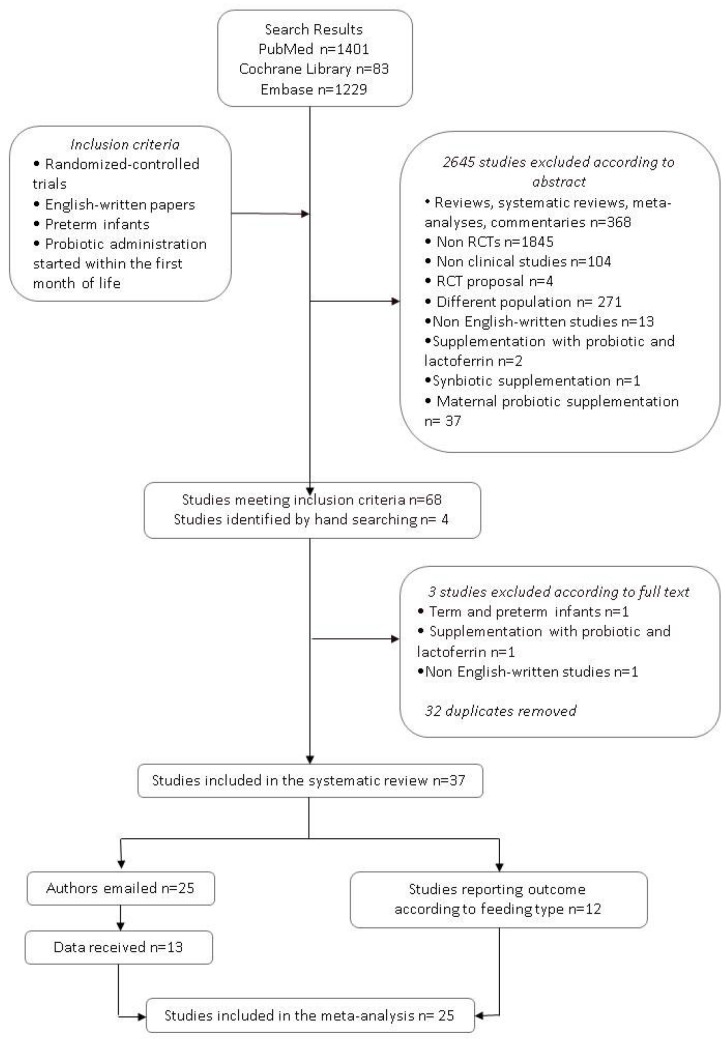
Flow chart of the search strategy and search results. The relevant number of papers at each point is given.

As shown in Figure 1, 68 papers met the inclusion criteria (35 in PubMed, 13 in the Cochrane Library search, and 20 in Embase). Four additional studies were identified by a manual search of the reference lists of included studies. Among these 72 studies, 32 were excluded as they were duplicates retrieved by at least two search engines. Three studies were excluded after examining the full texts: One study included both term and preterm infants [25], one study reported supplementation with probiotic plus bovine lactoferrin [26], and one study was not written in English [27].

Finally, 37 studies were eligible for the systematic review [28,29,30,31,32,33,34,35,36,37,38,39,40,41,42,43,44,45,46,47,48,49,50,51,52,53,54,55,56,57,58,59,60,61,62,63,64]. Details of the included studies are reported in Table 1; excluded studies are described in Table 2.

For each included study, the LOS rate in the probiotic and in the placebo/control group is reported in Table 3. The study by Dutta et al. [37] was reported three times, as it included three groups of patients supplemented with a probiotic given at three different doses. Data from the study of Hays et al. [39] were reported three times because three different interventions (*Bifidobacterium lactis* alone, *Bifidobacterium longum* alone, and *B. lactis* plus *B. longum*) were evaluated. The study by Romeo et al. [53] was reported twice, as it compared two different probiotics to placebo (*Lactobacillus reuteri* ATCC 55730 and *Lactobacillus rhamnosus* ATCC 53103), and the one by Tewari et al. [62] was reported twice because its participants were stratified as very preterm and extremely preterm.

Among the eligible studies, only twelve reported LOS according to feeding type during the study period: Eight studies reported LOS in exclusively HM-fed infants, either own mother’s milk (OMM) or donor human milk (DHM) [30,43,44,46,55,57,60,62], while four studies included exclusively formula-fed infants [31,32,61,64].

The corresponding authors of the remaining twenty-five studies were contacted by e-mail: data were provided for thirteen studies [28,36,37,38,39,42,49,50,51,52,56,58,59].

Twenty-five [28,30,31,32,36,37,38,39,42,43,44,46,49,50,51,52,55,56,57,58,59,60,61,62,64] studies were finally suitable for inclusion in the meta-analysis.

### 3.2. Probiotic and LOS: Overall Population

**Figure 2:**
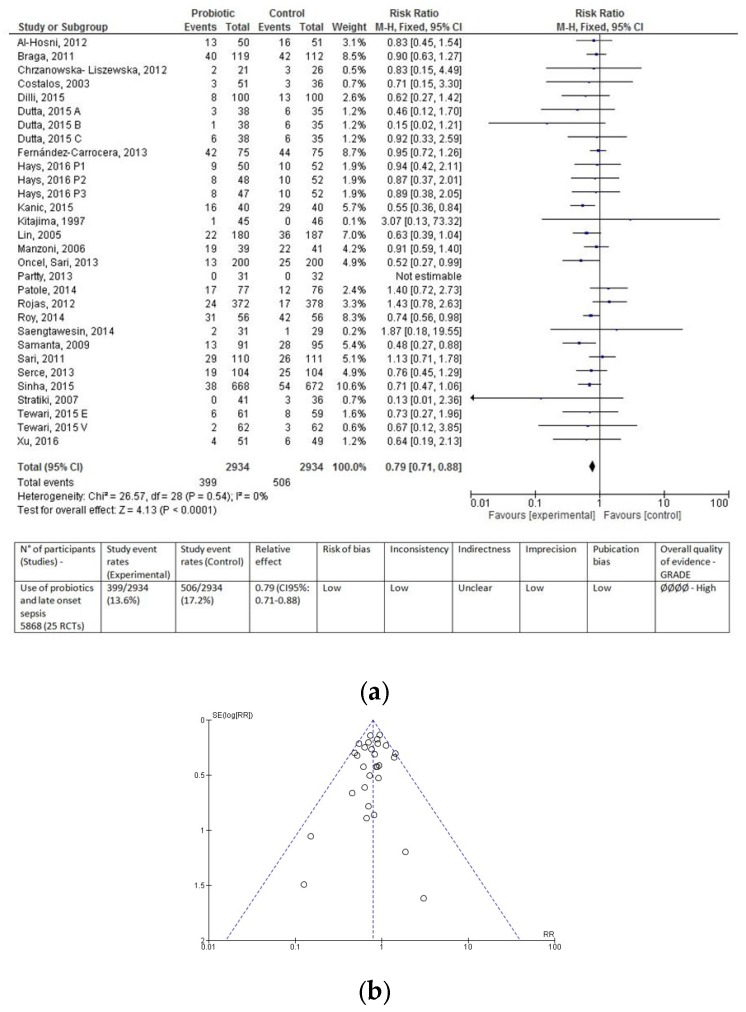
Forest plot (**a**) and funnel plot (**b**) of the included studies. The forest plot shows the association between the use of probiotics and late onset sepsis in the overall population of preterm infants. The evaluation of the overall results of the meta-analysis according to the GRADE approach is reported below the forest plot. The funnel plot does not show any clear visual asymmetry. M–H: Mantel–Haenszel method; RR, risk ratio; CI, confidence interval.

Overall, data from 5868 infants (2934 in the probiotic group and 2934 in the control group) were evaluated. Regardless of type of feeding, fewer infants in the probiotic group developed LOS compared to infants in the control group (399 (13.60%) vs. 506 (17.24%), respectively). Probiotic supplementation resulted in a significantly lower incidence of LOS (RR 0.79 (0.71–0.88), *p* < 0.0001; Figure 2a). Number needed to treat was 28. In other words, 28 infants would need to receive probiotic supplementation in order to prevent one additional case of LOS. The funnel plot did not show any clear asymmetry (Figure 2b).

### 3.3. Probiotic and LOS According to Type of Feeding

The data were then analyzed according to type of feeding (exclusive HM, exclusive formula, or mixed feeding).

**Figure 3:**
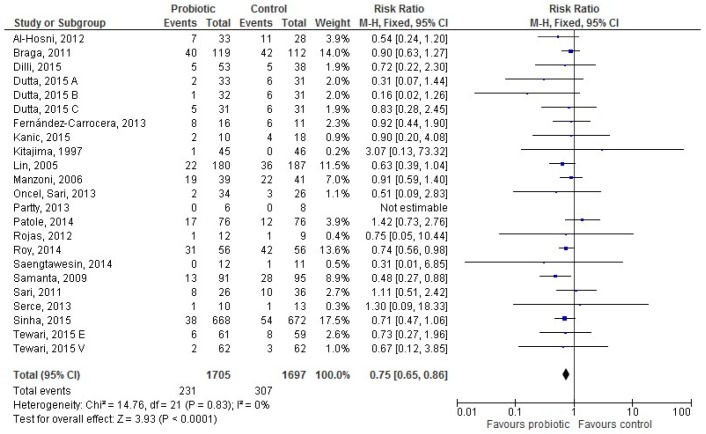
The forest plot shows the association between the use of probiotics and late onset sepsis in the twenty studies reporting data for exclusively human milk-fed preterm infants. M–H: Mantel–Haenszel method.

Twenty studies [28,30,36,37,38,42,43,44,46,49,50,51,52,55,56,57,58,59,60,62] provided data for 3402 exclusively HM-fed infants (1705 in the probiotic and 1697 in the control group). LOS occurred less frequently in HM-fed infants receiving probiotics than in controls (231 (13.55%) infants vs. 307 (18.09%), respectively); the RR was 0.75 ((95% CI 0.65–0.86), *p* < 0.0001), and heterogeneity among studies was absent (Figure 3).

**Figure 4:**
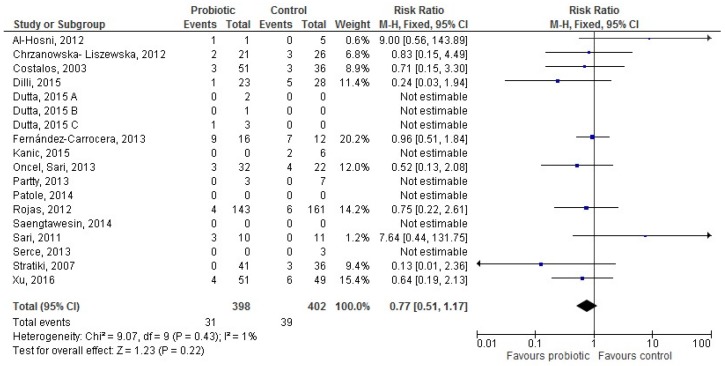
The forest plot shows the association between the use of probiotics and late onset sepsis in the sixteen studies reporting data for exclusively formula-fed preterm infants. M–H: Mantel–Haenszel method.

Sixteen [28,31,32,36,37,38,42,49,50,51,52,56,58,59,61,64] studies provided data for 800 exclusively formula-fed infants (398 in the probiotic and 402 in the control group). The difference in LOS incidence between groups was not significant (RR 0.77 (95% CI 0.51–1.17), *p* = 0.22; Figure 4).

**Figure 5:**
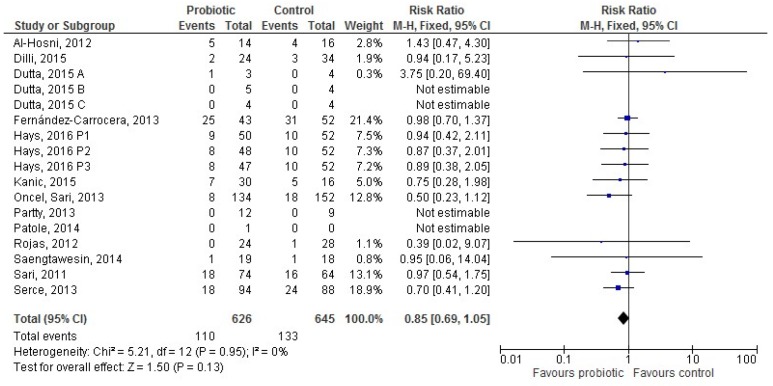
The forest plot shows the association between the use of probiotics and late onset sepsis in the thirteen studies reporting data for preterm infants receiving mixed feeding. M–H: Mantel–Haenszel method.

Thirteen [28,36,37,38,39,42,49,50,51,52,56,58,59] studies provided data for 1271 infants receiving mixed feeding (626 in the probiotic and 645 in the control group). The difference in LOS incidence between groups was not significant (RR 0.85 (95% CI 0.69–1.05), *p* = 0.13; Figure 5).

In order to examine in deeper detail the effect of probiotics in HM-fed infants, sub-meta-analyses restricted according to population and probiotic characteristics, as well as study quality, were performed.

#### 3.3.1. Population Characteristics: VLBW and Extremely Low Birth Weight (ELBW) Infants

Fifteen [28,36,38,42,43,44,46,49,51,52,56,57,58,59,62] studies reported data for 1516 exclusively HM-fed VLBW infants (760 in the probiotic and 756 in the control group). LOS occurred less frequently in infants given probiotics than in controls (114 (15%) infants vs. 151 (19.97%)), with an RR of 0.76 (95% CI 0.62–0.94; *p* = 0.01; *I*^2^ = 0%; fixed-effect model). 

Only two studies reported specific data on LOS in ELBW infants. One study [28] included only ELBW infants, who received exclusive HM or mixed feeding; the other one [62] recruited both VLBW and ELBW infants, who were exclusively HM-fed. In these studies, probiotic supplementation did not show any significant benefit in terms of LOS compared to a placebo.

#### 3.3.2. Probiotic Characteristics

Ten studies [28,30,37,38,42,44,55,56,57,60] reported data for 2560 HM-fed infants who received a probiotic mix (1281 infants) vs. placebo/no treatment (1279 infants). LOS occurred less frequently in infants given probiotics than in controls (169 (13.2%) infants vs. 242 (18.9%)), with an RR of 0.68 (95% CI 0.57–0.80; *p* < 0.00001; *I*^2^ = 0%; fixed-effect model).

Four studies [46,49,50,52] reported data for 175 HM-fed infants who received a single-strain *Lactobacillus* probiotic (91 infants) vs. placebo/no treatment (84 infants). No difference between groups in the incidence of LOS was documented (RR 0.87 (95% CI 0.58–1.32); *p* = 0.63; *I*^2^ = 0%; random effects model). *Lactobacillus* strains differed among studies: *Lactobacillus rhamnosus* was used in two studies [46,50] and *Lactobacillus reuteri* in two studies [49,52]. *Lactobacillus sporogenes* was used in one study [58], showing no differences between groups in LOS incidence; this latter study was not included in the pooled analysis, as *L. sporogenes* is a species which has not found international recognition, shows characteristics of both genera *Lactobacillus* and *Bacillus*, and its strain should be better classified as *Bacillus coagulans* [65].

Three studies [36,43,51] reported data for 334 HM-fed infants who received a single-strain *Bifidobacterium* probiotic (174 infants) vs. placebo/no treatment (160 infants). No difference between groups in the incidence of LOS was documented (RR 1.23 (95% CI 0.70–2.18); *p* = 0.47; *I*^2^ = 0%; random effects model). *Bifidobacterium* strains differed among studies: *Bifidobacterium breve* was used in two studies [43,51] and *Bifidobacterium lactis* in one study [36].

*Saccharomyces boulardii* was used in one study [59], as well as *Bacillus clausii* [62]: None of these studies showed a significant difference between infants treated with probiotics and controls in the incidence of LOS.

### 3.4. Methodological Study Quality

**Figure 6:**
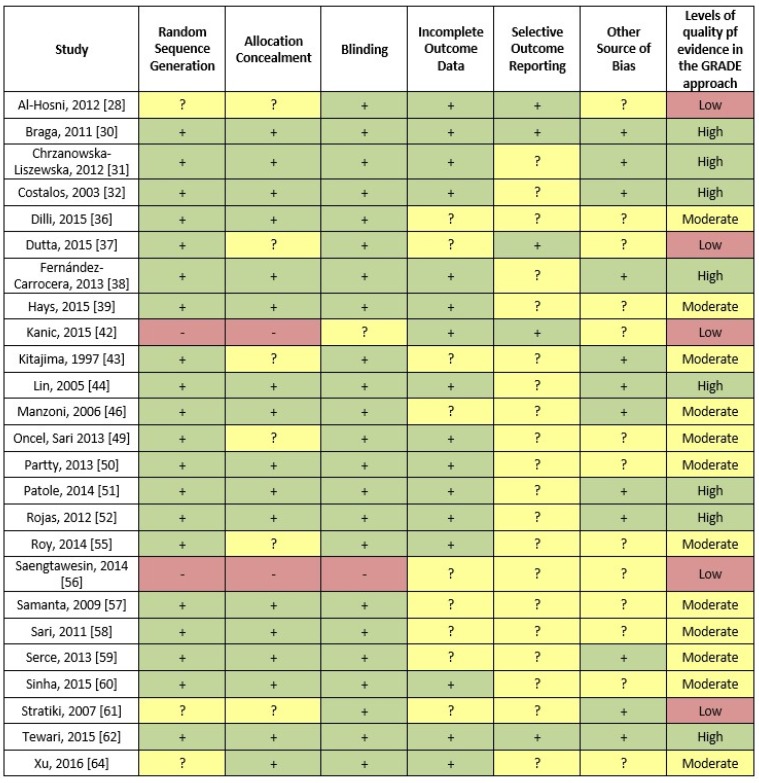
Evaluation of the quality of the studies included in the meta-analysis according to the risk of bias tool as proposed by the Cochrane collaboration (red represents a high risk of bias, yellow an unclear risk of bias and green a low risk of bias). In addition, the last column shows the assessment an assessment of the body of evidence using the GRADE working group approach.

The quality assessment of the studies included in the meta-analysis according to the risk of bias tool as proposed by the Cochrane collaboration is shown in Figure 6. The last column of the Figure also shows the assessment of the body of evidence using the GRADE working group approach.

Following a methodology similar to that used in the meta-analysis by Rao et al. [15], we conducted a sensitivity analysis including only studies which had a low risk of bias in both random sequence generation and allocation concealment. Sixteen studies [30,31,32,36,38,39,44,46,50,51,52,57,58,59,60,62] were included and reported data for 4628 infants (2306 in the probiotic and 2322 in the control group). The results were similar to those of the overall meta-analysis: LOS occurred less frequently in infants receiving probiotics than in controls (309 (13.4%) infants vs. 366 (15.76%)) with an RR of 0.85 (95% CI 0.75–0.97; *p* = 0.02; *I*^2^ = 0%; fixed effect model).

## 4. Discussion

In line with the results of previous papers [15,16], the present meta-analysis showed an overall benefit of probiotic supplementation for the prevention of LOS in preterm infants. However, when data were analyzed according to type of feeding, the beneficial effect of probiotics in reducing LOS was confirmed only in exclusively HM-fed preterm and VLBW infants, but not in infants receiving formula. Statistical heterogeneity among studies was almost absent and a low risk of publication bias was documented.

Two recent meta-analyses investigating the effect of probiotic supplementation on LOS in preterm infants reported an overall decrease in the risk of LOS in infants receiving probiotics compared to controls [15,16]. The studies included in the meta-analyses by Rao [15] and Zhang [16] are almost the same as those included in our updated systematic review; in the majority of the studies, both HM- and formula-fed infants were recruited, but no detailed data on the relationship between type of feeding and outcome were published.

Several data suggest that the impact of the type of feeding on clinical outcome in preterm infants is likely to be relevant [66]: It has been previously shown that HM feeding, per se, is associated with a reduction of the risk of developing LOS [67] and with a shorter time to achieve full enteral feeding in VLBW infants [68]. In addition, the use of probiotics in HM-fed, but not in formula-fed, infants appears to be related to a lower risk of NEC [19] and an earlier achievement of full enteral feeding [13].

It is plausible that the effect of probiotics on clinical outcomes could be mediated by HM properties [69]; actually, several HM components, including prebiotic HMOs, growth factors, immunological factors, and probiotic bacteria, can drive the establishment of a beneficial gut microbiota. In addition, HM can constitute the ideal soil for exogenous probiotics and promote a more effective crosstalk among probiotics, gut microbiota, and the developing immune system.

According to the latest recommendations, all preterm infants should receive exclusive HM; OMM is the best nutritional choice, and pasteurized DHM should be preferred to formula when OMM is not available or is contraindicated [70]. However, providing an exclusive HM diet to preterm infants presents a variety of challenges related to the prematurity itself and to hospitalization [71]. The term “exclusive HM feeding” may cover a range of feeding practices beyond direct breastfeeding, such as the use of fresh vs. frozen expressed breast milk given by bottle or tube feeding, the addition of HM fortifiers, and a variable duration of exclusive HM feeding. As described for pasteurization [72], some of these interventions might affect the nutritional and non-nutritional components of HM. In this perspective, the beneficial effect of probiotic supplementation in exclusively HM-fed infants might be related to a synergic action exerted by exogenous probiotics together with the prebiotic components of HM, which could partially restore the symbiotic potential of breast milk.

The data about exclusively HM-fed infants were analyzed according to population and probiotic characteristics in order to evaluate which preterm infants would benefit more from probiotic use and which probiotic strain or mixture of strains would be more beneficial. While there is evidence that probiotics are effective in reducing LOS in VLBW infants, no definite conclusion could be drawn for ELBW infants, as only two studies reported specific data on LOS in these infants, who remain the highest-risk and most vulnerable population.

The currently available literature does not provide a definite recommendation on which probiotic strain or mixture would be more effective in reducing LOS. In the 25 included studies, different probiotic strains and mixtures were used. Consistently with previous papers [12,16,73], our meta-analysis indicated that a mixture of different probiotic strains might be more effective in reducing LOS in exclusively HM-fed preterm infants. A possible explanation for this finding is that a probiotic mixture would provide a better ecological barrier and a more diverse immunological stimulation than a single strain.

The possible limitations of the present meta-analysis should be taken into consideration. Thirty-seven studies were potentially eligible for the meta-analysis, but only 25 studies provided separated data according to feeding type. In addition, infants’ classification according to feeding type was not homogeneous across studies, and the meta-analysis had to rely on unpublished information provided by the authors themselves. Finally, although no statistical heterogeneity was found, the characteristics of probiotic administration (dose, duration, time of initiation, and probiotic micro-organisms) differed among the included studies.

More importantly, no separate data for OMM-fed and DHM-fed infants were available; as a result, it was not possible to clarify whether the “synergic” effect of HM and probiotics applies to both OMM and DHM. It remains also unclear whether HM feeding, either OMM or DHM, has a “dose and time-dependent” effect on probiotic supplementation, as reported for outcomes such as NEC [66].

Probiotics appear to be generally safe, but it has to be acknowledged that there are some reports about the occurrence of sepsis in preterm newborns potentially linked to probiotic supplementation [74]. None of the studies included in the systematic review reported any side effect related to probiotic administration.

## 5. Conclusions

According to the results of the present meta-analysis, probiotic supplementation reduces the risk of LOS in exclusively HM-fed preterm infants. An exclusive HM diet should be the gold standard for all preterm, VLBW infants. Since direct breastfeeding is almost impossible in this population, it is likely that manipulations of HM, including pasteurization, refrigeration, and administration by tube or bottle, could affect HM bioactive properties; in this context, the administration of exogenous probiotics could help in restoring, at least partially, HM symbiotic properties.

Future research should be aimed at clarifying the relationship between feeding practices and probiotic supplementation, and at addressing the choice of the most effective probiotic products to be used in exclusively HM-fed infants.

## Figures and Tables

**Table 1:** Studies included in the systematic review.

Author, Year	Study Details	Study Population	Intervention Specie	Placebo
			Dose (D)	
			Start of Treatment (S)	
			End of Treatment (E)	
Al-Hosni, 2012 [28]	P	Preterm infants with BW 501–1000g, appropriate for gestational age, and ≤ 14 days of age at time of feeding initiation	*Lactobacillus rhamnosus GG* *Bifidobacterium Infantis*	Extra milk
	DB		D: 0.5 × 10^9^ CFU each probiotic, OD	
	R		S: first enteral feeding	
	C		E: discharge or until 34 w postmenstrual age	
	Multic.			
Bin-Nun, 2005 [29]	P	Preterm infants with BW < 1500g, who began enteral feeding on a weekday	*Bifidobacterium infantis* *Streptococcus thermophileus* *Bifidobacterium bifidus*	HM or FM
	B		D: 0.35 × 10^9^ CFU each probiotic, OD	
	R		S: Start of enteral feeding	
	C		E: 36 w postconceptual age	
Braga, 2011 [30]	P	Inborn infants with BW 750–1499 g	*Lactobacillus casei* *Bifidobacterium breve*	Extra HM
	DB		D: 3.5 × 10^7^ CFU to 3.5 × 10^9^ CFU OD	
	R		S: Day 2	
	C		E: Day 30, NEC diagnosis, discharge, death whichever occurred first	
Chrzanowska-Liszewska, 2012 [31]	P	Preterm infants with GA< 32 w and BW> 1000g, who started enteral formula feeding before enrollment	*Lactobacillus rhamnosus*	MDX
	DB		D: 6 × 10^9^ CFU, OD	
	R		S: Day 0–3 of life	
	C		E: Day 42 of supplementation	
Costalos, 2003 [32]	P	GA 28–32 w No major GI problem Not receiving antibiotics Not receiving breast milk	*Saccharomyces boulardii*	MDX
	R		D: 1×10^9^ CFU BD	
	C		S: Non-specified	
			Median duration of probiotic supplementation: 30 days	
Costeloe, 2016 [33]	P	Preterm infants with GA 23–30 w No severe malformation or any GI tract Malformation	*Bifidobacterium breve* BBG-001	Corn starch
	B		D: 8.3–8.8 log10	
	R		S: 43.9 h (median age)	
	C		E: 36 weeks’ postmenstrual age or discharge	
	Multic.			
Dani, 2002 [34]	P	Infants with GA< 33 w or BW < 1500 g	*Lactobacillus rhamnosus* GG	MDX
	DB		D: 6×10^9^ CFU OD	
	R		S: First feed	
	C		E: Discharge	
	Multic.			
Demirel, 2013 [35]	P	Preterm infants with GA≤ 32 w and BW≤ 1500 g, who survived to feed enterally	*Saccharomyces boulardii*	None
	B		D: 5 × 10^9^ CFU OD	
	R		S: First feed	
	C		E: Discharge	
Dilli, 2015 [36]	P	Preterm infants with GA< 32 w and BW< 1500 g, born at or transferred to the NICU within the first week of life and fed enterally before inclusion	*Bifidobacterium lactis*	MDX powder
	DB		D: 5 × 10^9^ CFU	
	R		S: Beyond d7 after birth	
	C		E: Death or discharge (max 8 weeks)	
	Multic			
Dutta, 2015 [37]	P	Preterm infants with GA 27–33 w, < 96 h of age, tolerating milk ≥ 15 mL/kg/day No GI/life-threatening malformations No NEC/sepsis	*Lactobacillus acidophilus* *Lactobacillus rhamnosus* *Bifidobacterium longum* *Saccharomyces boulardii*	Potato starch, MDX, magnesium stearate.
	B		Total D: 10^10^ CFU (high dose) or 10^9^ CFU (low dose), BD	
	R		S: Age< 96 h	
	C		E: Day 14 (short course) or day 21 (long course)	
Fernandez-Carrocera, 2013 [38]	P	Preterm infants with BW< 1500g	*Lactobacillus acidophilus* 1 CFU/g *Lactobacillus rhamnosus* 4.4 × 10^8^ CFU/g *Lactobacillus casei* 1 × 10^9^ CFU/g *Lactobacillus plantarum* 1.76 × 10^8^ CFU/g *Bifidobacterium infantis* 2.76 × 10^7^ CFU/g *Streptococcus thermophilus* 6.6 × 10^5^ CFU/g	None
	DB		Total D: 1g powder OD	
	R		S: Start of enteral feeding	
	C		E: Non-specified	
Hays, 2015 [39]	P	Preterm infants with GA 25–31 w and BW 700–1600, AGA, admitted to hospital within day 7 of life, Who initiated enteral feeding before day 5. Infants with NEC ≥ IB were excluded No severe malformation No severe clinical or surgical condition	*Bifidobacterium lactis only* *Bifidobacterium longum only* *Bifidobacterium lactis* + *Bifidobacterium longum*	MDX
	DB		D: 10^9^ CFU each strain, OD	
	R		S: Non-specified	
	C		Duration: 4 weeks if GA≥ 29 w , 6 weeks if GA≤ 28 w or until feeding interruption for more than 72 h	
	Multic.			
Hikaru, 2012 [ 40]	P	Extremely preterm infants and VLBW infants No major gastrointestinal tract surgery or multiple anomalies	*Bifidobacterium breve*	None
	R		D: 10^9^ CFU, OD	
	C		S: Day of birth	
			E: Discharge	
Jacobs, 2013 [41]	P	Preterm infants with GA< 32 w and BW< 1500 g	*Bifidobacterium infantis* BB-02 300 CFU × 10^6^ *Streptococcus thermophilus* Th-4 350 CFU × 10^6^ *Bifidobacterium lactis* BB-12 350 CFU × 10^6^	MDX powder
	DB		Total D: 1 × 10^9^ CFU × 1.5 g maltodextrin powder OD	
	R		S: enteral feed ≥ 1 mL every 4 h	
	C		E: discharge or term corrected age	
	Multic.			
Kanic, 2015 [42]	P	Preterm infants with GA< 33 w and BW< 1500 g	*Lactobacillus acidophilus* *Enterococus faecium* *Bifidobacterium infantis*	None
	R		Total D: 0.6 × 10^7^ CFU, BD	
	C		S: Start of enteral feeding	
			E: Discharge	
Kitajima, 1997 [43]	P	Preterm infants with BW< 1500 g	*Bifidobacterium breve* YIT4010	Distilled water
	R		D: 0.5 × 10^9^ CFU OD	
	C		S: Within 24 h of life	
			Duration of probiotic supplementation: 28 days	
Lin, 2005 [44]	P	Infants with BW< 1500 g, who started to feed enterally and survived beyond day 7	*Lactobacillus acidophilus*	None
			*Bifidobacterium infantis*	
	B		D: ≥ 10^6^ CFU each probiotic (= 125 mg/kg), BD	
	R		S. Start of enteral feeding	
	C		E: Discharge	
Lin, 2008 [45]	P	Preterm infants with GA< 34 w and BW< 1500 g, who survived to feed enterally	*Lactobacillus acidophilus* NCDO 1746 *Bifidobacterium bifidum* NCDO 1453	None
	B		D: 1 × 10^9^ CFU each probiotic (= 125 mg/kg), BD	
	R		S: Day 2 of age	
	C		Duration: 6 weeks	
	Multic.			
Manzoni, 2006 [46]	P	Infants with BW< 1500 g, ≥ 3 days of life, who started enteral feeding with HM	*Lactobacillus rhamnosus* LGG	None
	DB		D: 6 × 10^9^ CFU/day	
	R		S: Day 3 of life	
	C		E: End of the 6th week or discharge	
Mihatsch, 2010 [47]	P	Preterm infants with GA< 30 w and BW≤ 1500 g	*Bifidobacterium lactis* BB12	Indistinguishable powder
	R		D: 2 × 10^9^ CFU/kg 6 times a day	
	C		S: Start of enteral feeding	
			E: Non-specified	
Millar, 1993 [48]	P	Preterm infants with GA≤ 33 w	*Lactobacillus* GG	None
	DB		D: 10^8^ CFU, BD	
	R		S: Start of enteral feed	
			Duration: 14 days	
Oncel, Sari, 2013 [49]	P	Preterm infants with GA≤ 32 w and BW≤ 1500 g, who survived to feed enterally	*Lactobacillus reuteri* DSM 17938	Oil base
	DB		D: 1 × 10^8^ CFU OD	
	R		S: First feed	
	C		E: Death or discharge	
Partty, 2013 [50]	P	Preterm infants with GA 32–36 w and BW> 1500 g	*Lactobacillus rhamnosus* GG	Microcrystal line cellulose and dextrose anhydrate
	DB		D: 1 × 10^9^ CFU	
	R		S: Day 1	
	C		E: OD until day 30, BD until day 60	
Patole, 2014 [51]	P	Preterm infants with GA< 33 w and BW< 1500 g	*Bifidobacterium breve* M16-V	Dextrin
	DB		D: 3 × 10^9^ CFU OD (1.5 × 109 CFU OD for newborns≤ 27 w until they reached 50 ml/kg/day enteral feeds)	
	R		S: Start of enteral feed	
	C		E: Corrected age of 37 w	
Rojas, 2012 [52]	P	Preterm infants with BW≤ 2000 g, hemodynamically stable, ≤ 48 h of age (regardless start of enteral feeding)	*Lactobacillus reuteri* DSM 17938	Oil base
	DB		D: 1 × 10^8^ CFU OD	
	R		S: Age≤ 48 h	
	C		E: Death or discharge	
	Multic.			
Romeo, 2011 [53]	P	Preterm infants with GA< 37 w and BW< 2500g, who reached stable enteral feeding within 72 h of life	*Lactobacillus reuteri* ATCC 55730, 1 × 10^8^ CFU OD	None
	R		*Lactobacillus rhamnosus* ATCC 53103, 6 × 10^9^ CFU OD	
	C		S: Within 72 h of life	
			E: After 6 w or at discharge	
Rougé, 2009 [54]	P	Preterm infants with GA< 32 w and BW≤ 1500 g, ≤2 w of age, without any disease other than those linked to prematurity, who started enteral feeding before inclusion	*Bifidobacterium longum* BB536 *Lactobacillus rhamnosus* GG BB536-LGG	MDX
	DB		Total D: 1 × 10^8^ CFU/day	
	R		S: Start of enteral feeding	
	C		E: Discharge	
	Bic.			
Roy, 2014 [55]	P	Preterm infants with GA< 37 w and BW< 2500 g	*Lactobacillus acidophilus* 1.25 × 10^9^ CFU *Bifidobacterium longum* 0.1250 × 10^9^ CFU *Bifidobacterium bifidum* 0.125 × 10^9^ CFU *Bifidobacterium lactis* 1 × 10^9^ CFU	Sterile water
	R		Total D: 0.5 g powder, BD	
	DB		S: Within 72 h of life	
	C		E: After 6 w or at discharge	
Saengtawesin, 2014 [56]	P	Preterm infants with GA≤ 34 w and BW≤ 1500g	*Lactobacillus acidophilus* 1 × 10^9^ CFU *Bifidobacterium bifidum* 1 × 10^9^ CFU	None
	R		Total D: 125 mg/kg BD	
	C		S: Start of enteral feeding	
			E: End of 6th w of supplementation or discharge	
Samanta, 2009 [57]	P	Preterm infants with GA< 32 w and BW< 1500g, Who started to feed enterally and survived beyond 48 h of life	*Bifidobacterium infantis* *Bifidobacterium bifidum* *Bifidobacterium longum* *Lactobacillus acidophilus*	None
	DB		D: 2.5 × 10^9^ CFU each probiotic, BD	
	R		S: Non specified	
	C		E: Discharge	
Sari, 2011 [58]	P	Preterm infants with GA< 33 w and BW< 1500 g, Who survived to feed enterally	*Lactobacillus sporogenes*	None
	B		D: 0.35 × 10^9^ CFU, OD	
	R		S: Start of enteral feeding	
	C		E: Discharge	
Serce, 2013 [59]	P	Preterm infants with GA≤ 32 w and GA≤ 1500g, who survived to feed enterally	*Saccharomyces boulardii*	Distilled water
	DB		D: 0.5 × 10^9^ CFU, BD	
	R		S: Start of enteral feeding	
	C		E: Discharge	
Sinha, 2015 [60]	P	Preterm infants with GA≥ 34 w and BW 1500–2500 g	*Streptococcus thermophiles* *Bifidobacterium breve* *Bifidobacterium longum* *Bifidobacterium infantis* *Lactobacillus acidophilus* *Lactobacillus plantarum * *Lactobacillus paracasei* *Lactobacillus delbrueckii spp bulgaricus*	MDX
	DB		Total D: 10 × 10^9^ CFU per day	
	R		S: Day 3 of life	
	C		Duration: 30 days	
	Bic.			
Stratiki, 2007 [61]	P	Preterm infants with GA 27–37 w, formula fed	*Bifidobacterium lactis*	None
	B		D: 2 × 10^7^ CFU/ g milk powder	
	R		S: Start of enteral feeding	
	C		E: Discharge	
Tewari, 2015 [62]	P	Preterm infants with GA< 34 w	*Bacillus clausii*	Sterile water
	DB		D: 2.9 × 10^9^ spores	
	R		S: D5 in asymptomatic, d10 in symptomatic infants	
	C		E: 6 w of life, discharge, death, LOS diagnosis, whichever occurred first	
Totsu, 2014 [63]	P	Infants with BW< 1500 g	*Bifidobacterium bifidum*	Dextrin
	DB		D: 2.5 × 10^9^ CFU, divided in two doses	
	CLR		S: Within 48 h after birth	
	C		E: Body weight 2000 g	
	Multic.			
Xu, 2016 [64]	P	Preterm infants with GA> 30 and BW 1500–2500 g, formula fed	*Saccharomyces boulardii*	None
	B		D: 10^9^ CFU/Kg , BD	
	R		S: Start of enteral feeding	
	C		E: 28^th^ day of life or discharge	

B: Blinded, BD: Twice a day, Bic: Bicentric, BW: Birth weight, C: Controlled, CLR: Cluster-randomized, CFU: Colony forming units, DB: Double-blinded, DM: Donor milk, g: Grams, FM: Formula, GA: Gestational age, GI: Gastrointestinal, h: Hours, HM: Human milk, HMF: Human milk fortifier, LOS: Late onset sepsis, M: Masked, MDX: Maltodextrin, Multic: Multicentric, NEC: Necrotizing enterocolitis, OD: Once daily, OMM: Own mother’s milk, P: Prospective, PFM: Preterm formula, R: Randomized, w: Weeks

**Table 2:** Studies excluded from the systematic review.

Authors, Year	Study Summary	Reason for Exclusion
Awad, 2000 [25]	Living vs. killed *Lactobacillus acidophilus* vs. placebo given to neonates admitted to the study NICU	Term and preterm infants included
Manzoni, 2009 [26]	Bovine Lactoferrin (BLF) alone or BLF plus *Lactobacillus rhamnosus* GG given to VLBW neonates	Supplementation with probiotic and lactoferrin
Ren B, 2010 [27]	*Bacillus clausii* and *Clostridium* (*butyricum*) San Chang Le Kang given to preterm infants (exact gestational age unclear)	Non English-written study

NICU neonatal intensive care unit, VLBW very low birth weight.

**Table 3:** Incidence of late-onset sepsis (LOS) in infants treated with probiotics and in control.

Author, Year	Previous LOS Rate	Number of Subjects	LOS in Probiotic Group	LOS in Control Group
Al-Hosni, 2012 [28]	Not stated	50 probiotic 51 control	13/50	16/51
Bin-Nun, 2005 [29]	Not stated	72 probiotic 73 control	31/72	24/73
Braga, 2011 [30]	Not stated	119 probiotic 112 placebo	40/119	42/112
Chrzanowska-Liszewska, 2012 [31]	Not stated	21 probiotic 26 placebo	2/21	3/26
Costalos, 2003 [32]	Not Stated	51 probiotic 36 placebo	3/51	3/36
Costeloe, 2015 [33]	15%	650 probiotic 660 placebo	73/650	77/660
Dani, 2002 [34]	Not stated	295 probiotic 290 placebo	14/295	12/290
Demirel, 2013 [35]	Not stated	135 probiotic 136 control	20/135	21/136
Dilli, 2015 [36]	Not stated	100 probiotic 100 placebo	8/100	13/100
Dutta, 2015 A [37]	Not stated	38 probiotic 35 placebo	3/38	6/35
Dutta, 2015 B [37]	Not stated	38 probiotic 35 placebo	1/38	6/35
Dutta, 2015 C [37]	Not stated	38 probiotic 35 placebo	6/38	6/35
Fernández-Carrocera, 2013 [38]	Not stated	75 probiotic 75 placebo	42/75	44/75
Hays, 2015 P1 [39]	Not stated	50 probiotic 52 placebo	9/50	10/52
Hays, 2015 P2 [39]	Not stated	48 probiotic 52 placebo	8/48	10/52
Hays, 2015 P3 [39]	Not stated	47 probiotic 52 placebo	8/47	10/52
Hikaru, 2012 [40]	Not stated	108 probiotic 100 control	10/108	22/100
Jacobs, 2013 [41]	23%	548 probiotic 551 placebo	72/548	89/551
Kanic, 2015 [42]	Not stated	40 probiotic 40 control	16/40	29/40
Kitajima, 1997 [43]	Not stated	45 probiotic 46 placebo	1/45	0/46
Lin, 2005 [44]	Not stated	180 probiotic 187 control	22/180	36/187
Lin, 2008 [45]	Not stated	217 placebo 217 control	40/217	24/217
Manzoni, 2006 [46]	Not stated	39 probiotic 41 control	19/39	22/41
Mihatsch, 2010 [47]	40%	91 probiotic 89 placebo	28/91	29/89
Millar, 1993 [48]	Not stated	10 probiotic 10 control	0/10	0/10
Oncel, Sari 2013 [49]	Not stated	200 probiotic 200 placebo	13/200	25/200
Partty, 2013 [50]	Not stated	31 probiotic 32 placebo	0/31	0/32
Patole, 2014 [51]	Not stated	77 probiotic 76 placebo	17/77	12/76
Rojas, 2012 [52]	28%	372 probiotic 378 placebo	24/372	17/378
Romeo, 2011 A [53]	Not stated	83 probiotic 83 control	1/83	9/83
Romeo, 2011 B [53]	Not stated	83 probiotic 83 control	2/83	9/83
Rougé, 2009 [54]	Not stated	45 probiotic 49 placebo	15/45	13/49
Roy, 2014 [55]	33%	56 probiotic 56 placebo	31/56	42/56
Saengtawesin, 2014 [56]	Not stated	31 probiotic 29 control	2/31	1/29
Samanta, 2009 [57]	Not stated	91 probiotic 95 control	13/91	28/95
Sari, 2011 [58]	Not stated	110 probiotic 111 control	29/110	26/111
Serce, 2013 [59]	19%	104 probiotic 104 placebo	19/104	25/104
Sinha, 2015 [60]	17%	668 probiotic 672 placebo	38/668	54/672
Stratiki, 2007 [61]	Not stated	41 probiotic 36 control	0/41	3/36
Tewari, 2015 E [62]	21%	61 probiotic 59 placebo	6/61	8/59
Tewari, 2015 V [62]	21%	62 probiotic 62 placebo	2/62	3/62
Totsu, 2014 [63]	Not stated	153 probiotic 130 placebo	6/153	10/130
Xu, 2016 [64]	Not stated	51 probiotic 49 control	4/51	6/49
